# Value creation in sport business in the age of AI: a multidimensional framework

**DOI:** 10.3389/fspor.2026.1927685

**Published:** 2026-09-22

**Authors:** Hans Westerbeek

**Affiliations:** 1 Victoria University, Melbourne, VIC, Australia; 2 Victoria University Institute for Sustainable Industries & Liveable Cities, Melbourne, VIC, Australia

**Keywords:** artificial intelligence, competitive integrity, conceptual framework, legitimacy, social licence, sport value

## Abstract

Sport organisations, governments, sponsors, scholars, and athletes routinely appeal to the value of sport, and they almost always mean different things. The cost of this incoherence is no longer theoretical. Artificial intelligence is now a central subject of strategic, governance, and ethical debate in sport, yet the field lacks an integrative framework to assess what AI does to the value sport creates. This paper proposes a multidimensional framework — the Sport Value Architecture — that holds value plurality, capture asymmetry, and legitimacy in a single conceptual structure, and extends it to the age of AI. Sport generates three logically distinct types of value: competitive, commercial, and social. They stand in a determinate but non-uniform order: competitive value is a strict precondition of commercial value, and it conditions, rather than founds social value, which can arise from participation alone but collapses once a claimed contest is exposed as corrupted. Each is captured asymmetrically across four stakeholder groups, anchored in an irreducible sport activity accessible through three modes (Play, Spectate, Use), conditioned by three enabling conditions (governance, environmental responsibility, equity), and located within four exogenous force fields. Legitimacy is the outcome of the model working under defensible conditions, not a fourth deliverable. AI runs through every layer simultaneously: generating value within each value type, reshaping capture across every stakeholder group, stressing every enabling condition, and pressing on the irreducible activity itself. The framework supplies the conceptual apparatus to organise the strategic, governance, and ethical decisions this moment demands.

## 1 Introduction

The value of sport is appealed to constantly and almost never with shared meaning. Sport organisations claim it to defend broadcast rights deals, governments claim it to justify public investment, sponsors claim it to legitimise commercial activation, scholars claim it across half a dozen disciplinary traditions, and athletes claim it when they ask to be paid what they are worth. The same noun is doing radically different work in each of those sentences. The cost of this incoherence is felt in strategy that misfires, in policy that cannot defend its own grounds, in commercial decisions that erode the asset being monetised, and in social licence claims that do not survive scrutiny. Sport business has grown too large, too commercially exposed, and too publicly contested for the field to continue without an integrative model of what value sport creates, for whom, and under what conditions.

That gap is now also an AI problem. Artificial intelligence has become one of the most consequential subjects of strategic, governance, and ethical debate in sport in at least a generation. Performance AI is reshaping the contest. Generative AI is reshaping fan engagement and athlete branding (1, 2). Dynamic pricing AI is reshaping access. Algorithmic recommendation systems are reshaping fandom itself. Talent identification AI is reshaping the pathway from grassroots to elite. And the regulatory environment — anchored in instruments such as the European Union's AI Act — is reshaping what can lawfully be deployed at all. None of these developments can be assessed coherently inside a single value tradition. They cut across competitive, commercial, and social value at the same time, and they place pressure on the governance, environmental, and equity conditions that determine whether the resulting value remains legitimate. Without an integrative framework, AI decisions in sport are made one tool at a time, one rationale at a time, and one stakeholder at a time. That is not a sustainable basis for the next decade of sport business.

General management has supplied candidate frameworks. The triple bottom line (3) and its later self-critique (4), creating shared value (5) and the established critique of that concept (6, 7), and the family of ESG frameworks have all become standard vocabulary in sport boardrooms. None of them was built for sport, and each of them underspecifies the foundation on which sport rests. The triple bottom line treats environmental and social outcomes as parallel deliverables to economic ones; in sport they are not parallel, they are enabling conditions of the central deliverable. Creating shared value treats legitimacy as something strategic intent can produce; in sport, even shared value is itself derivative. There is no shared value to share unless the sporting contest is credible and the event is fair. The condition for shared value in sport is the integrity of the contest, not the strategic posture of the firm.

This paper proposes a sport-specific integrative framework — the Sport Value Architecture — and extends it to the age of AI. The framework rests on three pillars. The first is that without the sport activity there is no sport business. The activity is irreducible, and competitive value inherits that priority to different degrees for the two value types that follow from it: competitive value is a strict precondition of commercial value, since there is nothing to monetise without a contest, but its relationship to social value is conditional rather than absolute — social value can arise from participation independent of competitive intensity, though any social-value claim anchored in a contest collapses if that contest's integrity fails. AI sharpens this point by pressing on what makes a contest count as a contest in the first place. The second is that sport value is plural rather than unitary. Competitive, commercial, and social value are categorically distinct, each with its own mechanism of creation and its own conditions of integrity, and AI demonstrates this distinctness by generating value across all three simultaneously. The third is that legitimacy is the outcome of the framework working under defensible enabling conditions, not a fourth type of value to be delivered. Governance quality, environmental responsibility, and equity of access are the floor on which value creation rests, and AI stresses all three at the same time. Treating any of these conditions as a deliverable rather than as the conditions of deliverability is a category error, and AI makes the cost of that error large and immediate.

A brief note on method follows from the narrative-review approach announced above. Literatures were drawn in as the argument required them rather than through a pre-registered search protocol: sport economics and service-dominant logic to ground the activity and its modes, stakeholder theory and organisational legitimacy to ground capture and its defensibility, and the AI-ethics and AI-governance literatures to ground the extension. The framework's four components were not derived from a single parent theory; each was retained because it addresses a distinct failure the field has not yet held in one place — value conflation, capture opacity, legitimacy treated as a deliverable, and technological over-attribution — and each would leave that failure unaddressed if dropped.

The paper is structured as follows. Section 2 establishes the irreducible foundation: the sport activity and the three modes through which it generates value. Section 3 sets out the three logically distinct types of value, the mechanisms that produce them, and the way AI now runs through each. Section 4 develops the stakeholder grid, including an AI-extended version that exposes where value is captured under AI conditions. Section 5 makes the legitimacy-as-outcome argument, with particular attention to how AI stresses every enabling condition simultaneously. Section 6 locates the system inside its wider environment of context forces. Section 7 synthesises the AI through-line and proposes a six-question diagnostic for any AI decision in sport. Section 8 stress-tests the framework beyond elite professional sport. Sections 9 and 10 set out implications and conclude. The framework does not measure value, adjudicate trade-offs, or predict outcomes. It clarifies the conceptual terrain on which all of those tasks — including AI governance, strategy, and ethics — must be conducted. That clarity is the contribution.

## 2 The irreducible foundation: play, spectate, use

Sport business is a derivative industry. It does not generate its own subject matter. Whatever sport organisations, sponsors, broadcasters, platforms, or governments do, they do on the back of an activity that takes place when somebody plays, somebody watches, and somebody puts the resulting attention and meaning to use. Strip the activity and the rest dissolves. This is not a sentimental claim about sport's authenticity. It is a structural claim about where the value comes from. There is no broadcast right to sell if there is no contest to broadcast. There is no sponsorship to activate if there is no audience to reach. There is no social value to claim if there is no participation to point to. The sport activity is not one input among many. It is the input from which every other value-creating move in sport is derived.

The activity is accessible through three modes (8, 9). The first is Play: participants engage in the contest directly, whether as elite professionals, amateurs in organised competition, or recreational participants in non-competitive forms of the activity. The second is Spectate: audiences experience the contest through witnessing it, live in the venue, mediated through broadcast and digital platforms, or asynchronously through highlights, analysis, and conversation. The third is Use: organisations, brands, governments, and other institutions instrumentalise the attention and meaning the contest generates for purposes external to the contest itself — to sell, to brand, to teach, to develop, to influence, to govern. Each mode generates different value types, for different stakeholders, through different mechanisms. Each mode also presupposes the existence and integrity of an actual contest. Spectate has nothing to witness if there is no contest. Use has nothing to instrumentalise if there is no contest and no attention. Play is the contest. The three modes are how the activity becomes the source of value; they are not substitutes for it.

The foundational precedent for this kind of analysis in the sport management literature is Woratschek, Horbel and Popp’s (10) Sport Value Framework. Their framework, anchored in service-dominant logic (11, 12), correctly places sporting activities at the centre of analyses in sport management, treats sport events as platforms for value co-creation among multiple actors, and proposes a set of foundational premises for the discipline. It is the right starting point. This paper builds on that foundation and takes it further and deeper in four ways. First, it specifies three logically distinct value types — competitive, commercial, and social — each with its own mechanism of creation and its own conditions of integrity, rather than treating value as a generalised co-creation process. Second, it names the three modes (Play, Spectate, Use, see also (9) through which the activity becomes the source of value, making the relationship between activity and value structurally explicit. Third, it treats legitimacy as the outcome of the framework working under defensible enabling conditions, not as a co-created premise or a fourth domain to be delivered. And fourth, it stress-tests the framework beyond Western professional sport, in grassroots, Global South, and Indigenous and traditional sport contexts, to demonstrate that its logical structure is portable because it is anchored in the activity rather than in any particular commercial form.

## 3 Positioning the framework: gap, assumptions, and comparative contribution

The comparative contribution can be stated precisely. General management supplies frameworks that hold value together with legitimacy — the triple bottom line, creating shared value, stakeholder theory — but none was built for an industry whose central product is not, in the ordinary sense, a good or service; it is a contest whose outcome cannot be guaranteed without destroying the product. The independent sport-governance literature confirms the gap from the other direction: governance research in sport remains organised around organisational, systemic, and political forms, with comparatively little work connecting governance to the value the contest itself protects (13). Sport management supplies a framework built for sport in Woratschek, Horbel and Popp’s (10) Sport Value Framework, extended above in four specific ways, but no existing framework — general or sport-specific — holds sport-specificity, value plurality with a determinate order, and legitimacy as an emergent floor in a single structure; that is the gap this paper fills.

Each borrowed framework fails, and is challenged, for a specific reason. The triple bottom line treats economic, social, and environmental performance as parallel ledger entries that license trade-off; in sport, governance, environmental responsibility, and equity are conditions of legitimate production, not tradeable columns, so the trade-off the triple bottom line licenses is the move that destroys legitimacy. Creating shared value assumes legitimacy is something strategic intent can manufacture; in sport the causal arrow runs the other way, since there is no shared value to create unless the contest is already credible. Stakeholder theory (14, 15) correctly identifies competing claims on an organisation but supplies no prior theory of value types or their order, so a stakeholder grid alone tells you who wants something without telling you what, in kind, they are competing over. Woratschek, Horbel and Popp’s (10) Sport Value Framework, extended above in four specific ways, is the exception that gets the unit of analysis right but does not differentiate value by type or separately theorise legitimacy. Table 1 sets out the comparison across four dimensions: unit of analysis, value categories, treatment of legitimacy, and intended application.

**Table 1:** Comparative positioning of the sport value architecture.

Framework	Unit of analysis	Value categories	Treatment of legitimacy	Intended application
Sport Value Framework (10)	The sporting event as a platform for co-creation	Undifferentiated value, co-created among multiple actors	Implicit in co-creation; not separately theorised	Sport management research generally
Stakeholder theory (14, 15)	The firm and its stakeholder relationships	Value creation and capture among stakeholders, generically defined	An assumed condition of sound stakeholder management; not derived from a prior activity	Any organisation, any sector
Creating shared value (5)	The firm's competitive strategy	Economic and social value, treated as jointly producible	An output of strategic intent; something the firm can manufacture	Corporate strategy, any industry
Triple bottom line (3)	The firm's reporting and accountability	Economic, social, and environmental performance, as parallel ledger entries	A reporting outcome, commensurable with the other two columns	Corporate sustainability reporting, any sector
Sport Value Architecture (this paper)	The sport activity and its stakeholder grid	Competitive, commercial, and social value — logically ordered, non-uniform	An emergent outcome of the model working under defensible floor conditions; not a column, not manufacturable	Sport strategy, governance, and (extended) AI decision-making

The present framework advances theory beyond Woratschek, Horbel and Popp (10) by adding value plurality and legitimacy-as-outcome to their correct unit of analysis, and beyond stakeholder theory, shared value, and the triple bottom line by supplying the sport-specific reason their assumptions do not transfer, rather than simply asserting that sport is different. It should also be distinguished from the author's own recent related publications, which are instances this structure organises rather than restatements of it: the systematic review of platform power and athlete branding (2) and the account of generative AI in fan engagement (1) document particular AI-driven developments within the commercial domain, while the present framework is the architecture within which those and equivalent developments in the competitive and social domains can be located and assessed against a common standard of legitimacy.

This is also where artificial intelligence makes the foundational move more urgent. The framework has not previously been required to specify what makes a contest count as a contest. The contest could be taken as given, and the rest of the analysis proceeded from there. That is no longer adequate. Generative AI can produce highlights without the underlying contest taking place. AI can synthesise athletes, fabricate footage, and curate meta-competitions that look like sport without being constituted by the conditions sport requires. The Enhanced Games launched in Las Vegas in 2026 (16) is one adjacent example of how easily the form of a contest can be reproduced while the conditions that make a contest a contest are stripped away. The framework now has to answer the question it could once leave implicit: what makes a contest real, and therefore capable of generating value at all?

The answer is given by the integrity conditions of competitive value, set out in the next section. A contest is real when humans compete under uncertainty, under enforced rules, with meaningful stakes, and the outcome is the product of their performance. AI tools that protect those conditions strengthen the activity. AI tools that simulate, substitute, or counterfeit them dissolve it. The same logic that establishes the irreducible foundation also establishes the criterion by which any AI deployment in sport must be judged. The activity is not a constraint on what AI can do; it is the basis on which AI can do anything in sport at all.

## 4 Three logically distinct types of value

Sport generates three categorically distinct types of value: competitive, commercial, and social. They are not gradations of a single underlying construct, not three labels for the same phenomenon, and not interchangeable. Each has its own mechanism of creation, its own conditions of integrity, its own typical pattern of capture, and its own characteristic failure mode. The three sit in a determinate logical order, though the order is not uniform across the two derivative types. Competitive value is foundational to commercial value in an absolute sense: commercial value cannot exist where the sport activity does not exist, perform, and retain its integrity. Its relationship to social value is conditional: social value can be generated by participation alone, independent of competitive value, but where a social-value claim is made on the back of a contest, that claim is derivative of the contest's integrity and collapses with it. Section 3.4 sets out this distinction in full. The order is not aesthetic, and it is not normative; it is logical. AI does not reorder it. AI tests it.

One clarification is needed before the three types are examined in turn. The analysis that follows rests on a distinction that is foundational in the strategy literature but routinely blurred in sport: the distinction between creating value and capturing it (17, 18). Value creation is the generation of a benefit; value capture is the appropriation of that benefit by a particular actor. The two are separate operations, and the gap between them is where strategy, power, and distribution actually live. Sport is an unusually clear case of that gap, because the actors who create the value of the contest are rarely the actors who capture most of it. Artificial intelligence acts on both operations at once, and it does so in four directions that should be held apart. AI can advance value, extending what the activity is able to generate in the first place. It can enhance value, sharpening and deepening a benefit that already exists. But it can equally distort value, bending capture away from the actors whose participation creates it, and it can destroy value, dissolving the very conditions on which the benefit depends. Each of the three value types is examined below for all four effects, because AI seldom does only one of them at a time, and the same deployment that advances value on one measure can be destroying it on another.

### 4.1 Competitive value — the contest is the product

Competitive value is generated by the contest itself. The mechanism is the performance of the activity, the determination of the outcome, and the stakes attached to that outcome. Competitive value rests on four integrity conditions. The first is uncertainty of outcome (19–21): the result must not be known in advance. The second is authentic performance: real participants making real efforts with real consequences for themselves. The third is competitive integrity: rules that are fair and enforced consistently, with the contest protected from corruption, manipulation, and undisclosed enhancement. The fourth is meaningful stakes: the outcome must matter to the participants, to the audiences, and to the communities that hold the sport as part of their identity. Each of the four conditions is necessary. Stripping any of them dissolves competitive value, and with it the basis for commercial and social value derived from the contest.

The capture of competitive value runs across all four stakeholder groups. Athletes capture it in the form of achievement, recognition, status, and the career trajectories that authentic performance underwrites. Fans capture it through the experience of witnessing real contests with real stakes, and through the identities they build around those contests. Commercial partners capture it through the audience and the authenticity that an actual contest supplies for branding, sponsorship, and rights. Governments capture it through national pride, international prestige, and the talent infrastructure that the contest justifies.

The characteristic failure mode of competitive value is when the activity continues to look like sport but no longer is sport in the relevant sense. Match-fixing, undisclosed doping, manufactured competition, and scripted outcomes are the classical forms. The form remains; the substance does not. Fans understand this immediately, even when the regulators do not, and the social licence collapses in the order one would expect — first among the participants, then among the audiences, then among the partners, and finally among the regulators who are now responding to a crisis they could have prevented (22).

Artificial intelligence enters the competitive domain in two opposite forms. The first is AI that strengthens the integrity conditions: performance and load-management AI improve training, reduce avoidable injury, and extend elite careers; AI scouting expands the pool from which talent is identified; AI officiating, applied to clear factual questions such as line calls and goal-line decisions, increases the accuracy and consistency of rule enforcement. These applications protect the four conditions; they do not compromise them. The second form is AI that erodes them: generative AI substitutes synthetic athletes and fabricated footage for performance and the contest itself, opaque or biased algorithmic selection compromises the fairness of rule application, and AI integrated with gambling markets multiplies the points at which a contest can be manipulated for off-field gain. The Enhanced Games, with no testing and an openly pharmacological model, demonstrates how easily the conditions can be stripped while the surface form of the contest is preserved (16). The integrity conditions of competitive value are therefore also the primary test of any AI deployment that touches the contest: not whether the AI works, but whether it protects or compromises uncertainty, authenticity, integrity, and stakes. AI that fails this test does not create competitive value. It consumes it.

### 4.2 Commercial value — converting attention into economic return

Commercial value is generated by the monetisation of the attention and the meaning the contest produces. The mechanism is conversion: attention into audiences, audiences into rights, rights into revenue, revenue into reinvestment. The commercial domain is broad. It spans broadcast and sponsorship revenue, the direct-to-consumer and data businesses that now sit alongside them, and the design, manufacture, wholesale, and retail of the sporting goods and apparel that equip and clothe the activity — a production and distribution industry that is part of sport business even though it operates at one step removed from the contest. The integrity conditions of commercial value are different from those of competitive value. They require legible audiences that can be measured and addressed; transferable rights that can be contracted, owned, and traded; and a sustainable revenue logic that does not consume the conditions of competitive value on which the entire monetisation rests. The conditions are commercial, but the constraint is not. A monetisation strategy that destroys the contest destroys its own basis.

Commercial value in sport has evolved structurally rather than incrementally: from the gate-and-broadcast era of the 1960s–1980s, through the sponsorship and multi-platform era of the 1990s–2000s, to the direct-to-consumer and digital ecosystem era of the 2010s (9, 23, 24). The current era is defined by private equity ownership, multi-club portfolios, and the integration of AI-driven personalisation and athlete branding across the full value chain (1, 2).

The capture of commercial value is asymmetric. Rights-holders and platform owners capture the largest share. Athletes capture a contested share that has grown materially with the rise of athlete-owned digital channels and direct branding (25). Fans pay, in money and in attention; they do not capture commercial value, they supply it. Governments capture commercial value indirectly through taxation, employment, and infrastructure utilisation, but rarely in proportion to the public investment that made the commercial activity possible in the first place.

The characteristic failure mode of commercial value is extraction that consumes the conditions of competitive value. Private equity logic and multi-club portfolios optimise for cross-asset efficiency in ways that can erode the sporting decisions on which the contest depends. Over-monetisation alienates the communities whose identification with the sport is the source of the attention being monetised. Data exploitation strips athletes and fans of the autonomy and dignity that authentic participation requires. Gambling integration multiplies the surfaces on which the contest can be manipulated. The pattern is the same across each: commercial value extracted in a way that destroys the basis for further commercial value.

Artificial intelligence is the current vector of every one of these dynamics. Dynamic pricing AI extracts margin from fan demand at finer granularity than ever before and can systematically exclude the communities that built the club identity. Algorithmic personalisation deepens engagement but directs value capture toward the platforms that own the recommendation systems rather than toward the sport organisations that supply the content (1, 26). Generative AI lowers the cost of producing fan-facing content while concentrating the data assets and model capabilities that determine who can produce it at scale. AI-driven athlete branding redistributes value capture toward athletes who can build and operate direct platforms, away from the sport organisations that historically intermediated those relationships (2). The deep structural question that AI surfaces in the commercial domain is one of credibility as well as cash. Capital is increasingly cheap; credibility is increasingly scarce, and the latter is what the contest supplies (27). Commercial value in the age of AI is captured by those who own the data, control the algorithms, and retain the credibility of the contest. The first two can be bought. The third cannot.

### 4.3 Social value — the collective benefit and the basis of public investment

Social value is the collective benefit that sport produces beyond the transaction. The mechanism is participation, identity, collective experience, and the public-good outcomes that participation and identification underwrite. The domains in which sport produces social value are well established in the literature: physical health and disease prevention (28, 29); mental health and wellbeing; social cohesion and civic identity; diversity, inclusion, and representation; education, youth development, and life-skill formation; and peace, reconciliation, and international development (30–32). These are not aspirational claims. They are the outcomes that justify public investment in sport at scale and the basis on which sport's social licence rests.

The capture of social value is also distributed differently from competitive and commercial value. Communities and civil society capture it as collective good. Participants capture it directly through health, identity, and the social ties that participation produces. Governments capture it as fiscal saving, reduced burden on health systems, and social integration. Commercial partners capture it indirectly through reputational alignment and the corporate social responsibility positioning that participation in social value enables (33, 34). Social value is the part of the model where the distribution most often departs from the rhetoric. Social value is claimed widely and captured narrowly; the populations who are named as the beneficiaries are not always the populations who receive the benefit.

The characteristic failure mode of social value is when claims of social value function as cover for commercial extraction or political instrumentalisation. Sportswashing of communities, of governments, and of regimes operates by attaching credible social value claims to actors whose underlying conduct undermines those claims (35–37). The same dynamic appears in domestic forms whenever a sport organisation deploys community programs as legitimation for commercial decisions that exclude the very communities the programs are designed to reach. The form of social value is preserved; the substance is hollowed out.

Artificial intelligence enters the social domain in two directions, both consequential. The first is democratisation: AI-enabled coaching tools make elite-grade analysis available to grassroots clubs that could not previously afford it, accessible commentary and real-time translation expand who can participate as athlete, coach, or fan, mental wellness AI supports those for whom traditional clinical support is not accessible, and community sport analytics give grassroots organisations the participation and outcome data they need to compete for public and philanthropic funding. This democratising potential is real, and concentrated where the existing distribution of analytical capability is most unequal (1). The second direction is the opposite: AI built on biased training data reproduces the exclusions that have historically determined who participates and who advances, talent identification AI trained on well-resourced academies underweights athletes from under-resourced backgrounds, algorithmic pricing applied to participation or facility access prices out the populations the social-value claim is meant to serve, and extractive rather than reciprocal data practices export the value participation creates to entities that did not contribute to it. The social-value claim under AI is therefore a design claim: AI in the social domain is socially valuable only when training data, access guarantees, data reciprocity, and representation are made deliberately and verifiably.

### 4.4 The order matters — and AI does not change it

The three value types stand in a determinate logical order. Commercial value cannot exist where there is no contest to monetise. Social value can exist with very low competitive value — recreational participation produces real social value without an elite contest behind it — but where competitive value is claimed and is in fact corrupted, social value collapses with it, because the legitimacy of the claim depends on the legitimacy of the contest. This qualifies rather than contradicts the ordering claim stated earlier: the sport activity itself is the model's absolute foundation, competitive value is foundational to commercial value in that same absolute sense, and competitive value is foundational to social value only conditionally — where a social-value claim is anchored in a contest rather than in participation alone. Competitive value is the load-bearing element wherever a contest is claimed. The three types are not equally suspended in the air.

Artificial intelligence does not reorder the framework. AI that generates commercial value by dissolving competitive value destroys its own basis; the commercial value is real for one cycle and disappears in the next. AI that generates social value by hollowing out participation destroys the participation that the social-value claim depends on. The order holds, and AI is the test under which it holds. This is the conceptual content of the first pillar: without the sport activity there is no sport business, and any AI deployment that compromises the activity compromises the entire model that runs on top of it.

### 4.5 Where political and soft-power effects live — and how AI changes their speed and reach

Political and soft-power effects — state investment, prestige strategies, sport diplomacy, sportswashing — are real and consequential (35, 37), but the framework does not treat them as a fourth value type. They are emergent effects arising at the intersection of the contest's credibility (the integrity of competitive value), the stakeholder configuration engaged, and the context forces acting on that combination; treating them as a separate value type would obscure this interaction and the fact that such effects evaporate, or invert into reputational damage, when contest credibility or stakeholder alignment fails.

Artificial intelligence accelerates rather than restructures these dynamics. AI-driven content distribution and algorithmic recommendation systems compress the timescale of reputational accumulation and collapse alike, and deepfake-adjacent content and synthetic athlete personas multiply the surfaces on which such effects can be manufactured, defended, or attacked. The mechanism is unchanged; AI is the accelerant. The framework's diagnostic task remains keeping the analyst focused on the underlying interaction — contest, stakeholders, context — rather than the accelerated surface phenomenon.

Proposition 1. Competitive value is a necessary antecedent of commercial value: no commercial value can be legitimately sustained from a contest whose competitive integrity has failed.

Proposition 2. Competitive value conditions, but does not necessitate, social value: social value can be generated by participation independent of competitive intensity, but any social-value claim anchored in a contest collapses if that contest's integrity fails.

## 5 The stakeholder grid: capture, distribution, and tension

The three value types are categorically distinct, but the question that actually drives strategy, governance, and policy in sport is not which value type is being created. It is who receives it. Capture is where the analytical work happens, and the distinction between creating value and capturing it — foundational in strategy theory, and nowhere more visible than in sport — is the distinction the grid is built to expose (18). The same event — a single match, a single season, a single mega-event — creates competitive, commercial, and social value simultaneously, and the value is captured asymmetrically by stakeholders whose interests are not aligned. Strategy in sport is largely the practice of choosing whose interests to prioritise, when, and on what justification. Governance is the practice of holding those choices accountable to the populations that bear their consequences. The stakeholder grid is the device that makes both visible.

Four stakeholder groups carry the analytical load. The first is athletes and participants — those whose effort, performance, and bodies are the source of the contest. The category includes elite professionals, amateurs in organised competition, recreational participants, and the development pipeline that supplies all three. The second is fans and communities — those whose attention, identification, and emotional investment sustain the meaning of the contest. The category includes paying spectators, mediated audiences, the local communities whose identity is bound up with a particular sport organisation, and the diasporic and digital communities whose attachments operate at distance. The third is commercial partners — those whose capital, infrastructure, and operational capacity monetise the contest and its attention. The category includes sponsors, broadcasters, platform owners, rights-holders, equipment manufacturers, the sporting goods and apparel businesses that produce, wholesale, and retail the products of sport, sport organisation owners and investors, and the financial actors who increasingly position sport assets inside investment portfolios. The fourth is governments and civil society — those who fund, regulate, and host sport for public ends. The category includes national and subnational governments, intergovernmental bodies, regulators, the legal systems that hold sport organisations to account, and the civil society actors who pursue social outcomes through sport.

Cross-tabulating the four stakeholder groups against the three value types produces the central matrix of the framework (Figure 1), read as a single instrument rather than cell by cell. Athletes, for instance, capture competitive value as achievement and career trajectory, commercial value as wages and image rights, and social value as identity and wellbeing; the same logic of differentiated capture runs across fans and communities, commercial partners, and governments and civil society, each drawing a distinct form from each of the three value types, as Figure 1 sets out in full. It is the cross-tabulation, not any individual cell, that is the contribution.

**Figure 1:**
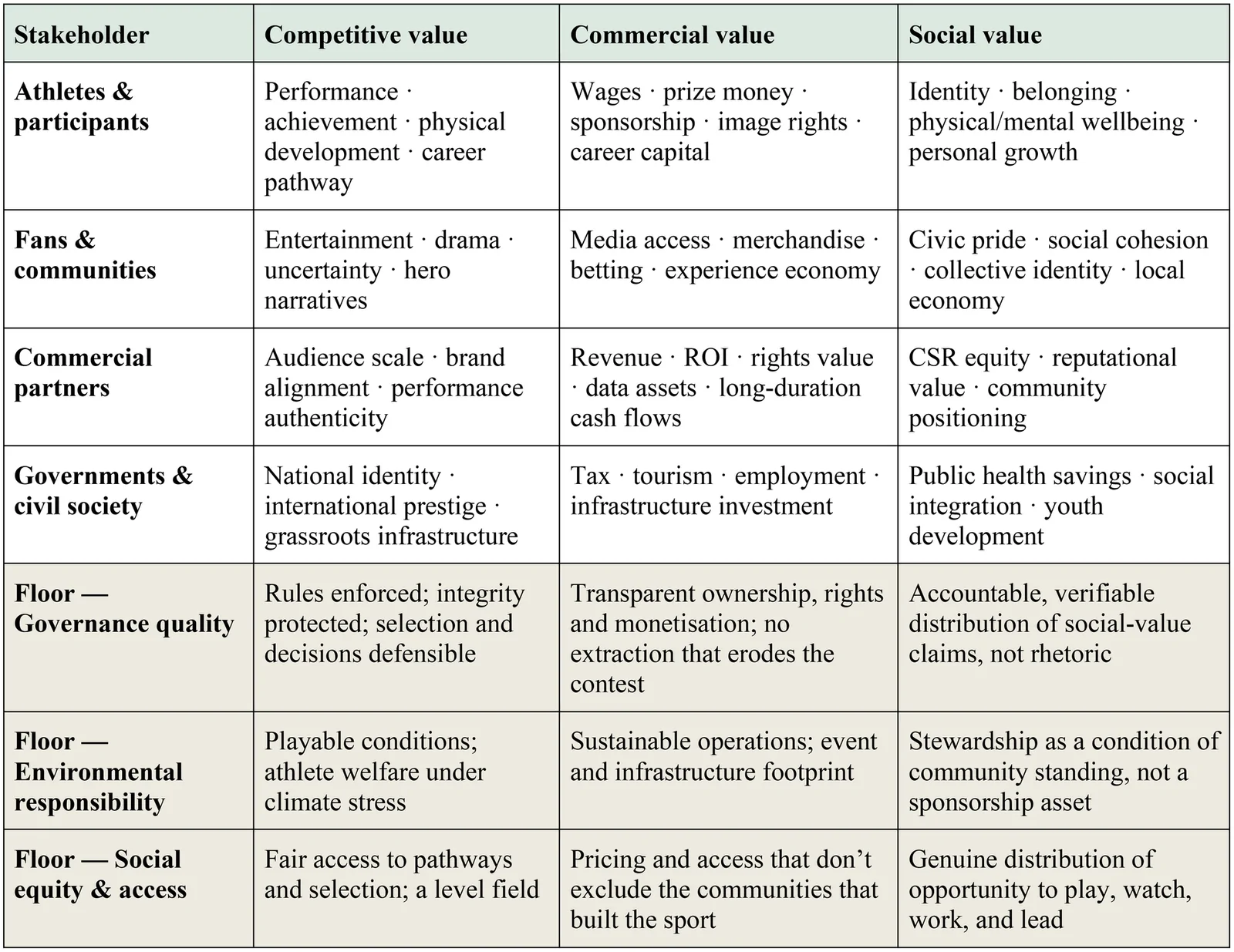
The sport value architecture matrix (base 4 × 3).

The matrix exposes three things that are routinely left implicit in sport business discourse. The first is capture asymmetry: who actually receives each form of value, in what form, and on what timescale. The asymmetry is structural and predictable. Commercial partners capture commercial value disproportionately. Fans and communities supply attention and pay for it, and capture the experiential and identity dimensions that the contest produces. Athletes capture a contested share of commercial value and the full burden of competitive performance. Governments and civil society capture social value indirectly through fiscal saving and political legitimation, but rarely capture commercial value in proportion to the public investment that made commercial extraction possible. The asymmetry is not a problem; it is the structure. The question for governance is whether the asymmetry is defensible.

The second is strategic tension: where one stakeholder group's value gain comes at another group's expense. Commercial partner maximisation through premium pricing and corporate hospitality systematically excludes the communities whose identity sustains the sport. Private equity and multi-club ownership logic, optimising for cross-asset portfolio efficiency, erodes the sporting decisions on which the competitive integrity of the contest depends. Government public investment underwrites the infrastructure and talent pipelines that make commercial value extraction possible, while the resulting commercial value is captured by private actors. The enabling conditions of governance, sustainability, and equity are framed as constraints on the commercial model by the actors who would prefer not to bear their cost, when in fact they are the conditions on which the commercial model rests. These tensions are not problems to be solved. They are the strategic and ethical landscape in which sport operates. The framework's contribution is to make them visible so that the decisions taken across them can be defended on their merits.

The third is the governance question: whose value claim is prioritised, on what basis, and with what accountability to the populations that bear the consequences. The governance question is also where stakeholder theory does its analytical work (14, 38). It is sharpened, conceptually, by the broader scholarship on value pluralism, particularly Boltanski and Thévenot’s (39) account of the multiple orders of worth that coexist inside any economic and social arrangement, and Stark’s (40) treatment of the productive friction that arises when those orders are held in view at the same time. Sport is a paradigm case of plural worth. The framework does not collapse the orders; it makes them legible, and it makes the trade-offs across them subject to the same kind of justification that strategy and governance routinely demand of other distributional decisions.

Artificial intelligence reshapes capture across every cell of the matrix, producing a second matrix that has to be read alongside the first (Figure 2). Athletes, for instance, gain capture in the competitive domain through AI injury prevention and personalised training, in the commercial domain through direct athlete-branding channels that redirect capture away from traditional intermediaries (2, 25), and in the social domain through democratised access to analytics previously available only to the elite. The same redistribution — competitive, commercial, and social capture reshaped by AI-specific mechanisms — runs across fans and communities, commercial partners, and governments and civil society, as Figure 2 sets out in full.

**Figure 2:**
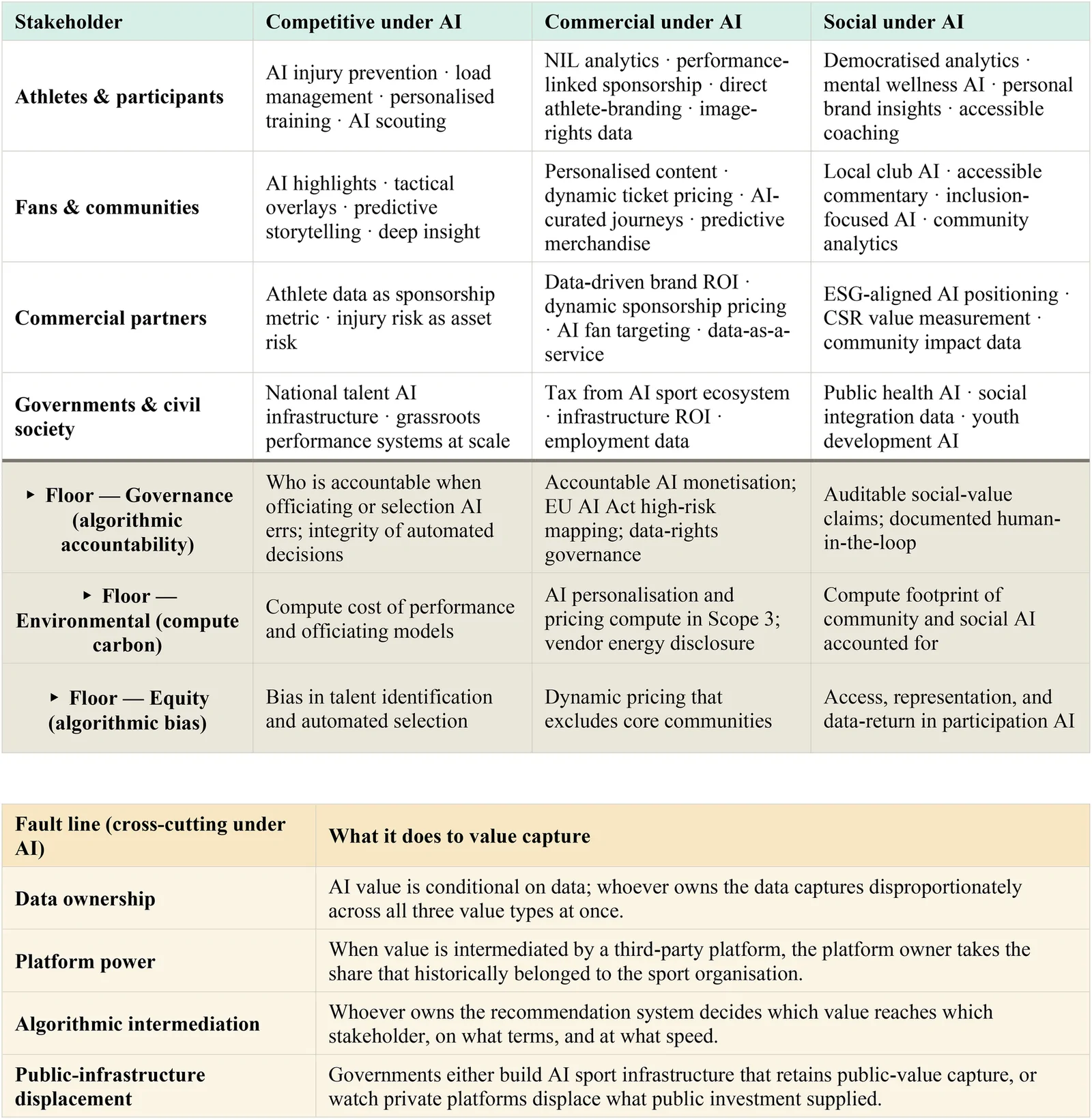
The AI-extended sport value architecture matrix.

The AI-extended matrix is not simply the original matrix with more entries in it. It surfaces a set of structural tensions that the original matrix did not have to resolve. The first is data ownership: AI value is conditional on data, and the actor that owns the data captures disproportionate value across all three value types simultaneously. The second is platform power: when value is intermediated by a third-party platform, the platform owner captures a share that historically belonged to the sport organisation or to the stakeholder generating the underlying activity, a dynamic consistent with the broader account of platforms as infrastructural intermediaries that extract value from the transactions they convene (1, 41). The third is algorithmic intermediation: the entity that owns the recommendation system determines which value reaches which stakeholder, on what terms, and at what speed. The fourth is public infrastructure displacement: governments either build AI sport infrastructure that retains public-value capture, or watch private platforms displace the public infrastructure that public investment historically supplied. These are not new tensions in kind; they are tensions of the kind the original framework already exposes. They are sharpened by AI, and the AI-extended matrix is the device that makes the sharpening visible.

Proposition 3. Capture asymmetry across the four stakeholder groups is structural rather than incidental, and artificial intelligence redistributes but does not eliminate it: capture shifts toward whichever actor owns the data, the platform, or the algorithm mediating a given exchange, regardless of which stakeholder group originally created the underlying value.

## 6 Enabling conditions: why legitimacy is not a fourth type of value

The framework so far has identified three value types and four stakeholder groups and has shown how artificial intelligence reshapes capture across all twelve cells of the resulting matrix. It has not yet addressed the most consequential conceptual move of the framework, which is the treatment of legitimacy. Legitimacy is the question on which sport organisations, governments, sponsors, and regulators most frequently fail, and it is the question on which the existing tri-partite frameworks of general management most clearly underspecify what sport requires.

Three enabling conditions sit beneath every cell of the stakeholder-value matrix. The first is governance quality: transparent, accountable, rule-bound institutions whose decisions can be defended to the populations affected by them. The second is environmental responsibility: operational, infrastructural, and event-level practices that are compatible with the sustainability commitments sport organisations have made and the planetary conditions that sport ultimately depends on. The third is social equity and access: the fair distribution of opportunities to play, watch, work in, and lead sport across the populations sport claims to serve. The three conditions are not value types. They are the floor on which legitimate value creation stands, and without it, it fails. They cannot be delivered to a stakeholder. Governance cannot be handed to a community in the way entertainment is. Equity cannot be sold to a market in the way broadcast rights are. Sustainability cannot be packaged as a sponsorship asset in the way audience reach is. Treating any of the three as a deliverable rather than as the conditions of deliverability is a category error, and it is the error that the triple bottom line, ESG, and shared value frameworks have repeatedly induced when imported into sport.

The distinction is worth stating with care, because it is the philosophical hinge of the framework and the point on which it parts company with the dominant value models of general management. The triple bottom line (3) treats economic, social, and environmental performance as three parallel ledger entries: commensurable quantities that license trade-off, so a deficit on one can in principle be offset by a surplus on another. That logic may hold for a firm whose product is genuinely independent of its social and environmental conduct; it does not hold in sport, for a structural rather than a moral reason. Governance, environmental viability, and equity of access are not co-products standing alongside the value the activity generates — they are the conditions under which that value can be generated legitimately at all. A contest run under captured governance, on an unplayable planet, or for a public systematically shut out of it does not yield a smaller quantity of legitimate value to net against revenue; it yields value whose legitimacy has failed, and within this model failed legitimacy cannot be offset, because there is no positive column for it to be set against. A column is a quantity of value that you can trade; the floor is a level below which the whole structure ceases to stand — which is why the three (floor) conditions sit beneath the matrix rather than inside it as a fourth column. Even the most generous general-management position, that social and environmental performance are merely on par with the economic, concedes too much: parity still implies commensurability, and commensurability still implies exchange. In sport the social and environmental are prior to the economic, not parallel with it. Artificial intelligence turns this from a conceptual nicety into an operational emergency, because it degrades the conditions — through opaque decision-making, compute emissions, and embedded bias — faster and more invisibly than any prior technology, and faster than trade-off logic is equipped to notice.

Legitimacy is the outcome of the three value types being created and distributed under defensible enabling conditions. It is not produced by strategic intent; it is earned by the model working under conditions that can be defended to the populations affected by it. It is observed in the behaviour of the actors whose recognition determines whether sport organisations retain their public standing — athletes who are willing to play, fans who are willing to attend, communities who are willing to host, governments who are willing to fund, partners who are willing to associate. It collapses when any of those actors withdraws recognition, and it is observable when it collapses more reliably than when it holds. Suchman’s (42) account of organisational legitimacy as a composite of pragmatic, moral, and cognitive recognition, updated by Deephouse, Bundy, Tost, and Suchman (43) into a multilevel process of evaluation by individual and collective judges, supplies the underlying mechanism. The social licence to operate, developed first in the extractive industries and now widely applied across sectors (44, 45), supplies the practical vocabulary in which the recognition operates. Legitimacy in sport is the joint outcome of competitive, commercial, and social value being produced and distributed in a manner that does not violate the enabling conditions on which the model rests.

The implication for sport governance and integrity is direct. Governance reform that produces the appearance of accountability without the substance of it does not produce legitimacy; it produces legitimacy debt, accumulated against the day the substance is tested (46, 47). Environmental commitments that hit reporting metrics while leaving operational practice unchanged do the same. Equity policies that increase the visibility of underrepresented groups without changing the distribution of opportunity also do the same. The framework supplies the language to name this: an enabling condition is met when the substance is verifiable in the practice, not when the metric is reported. The social licence does not negotiate.

Each enabling condition is examined in turn below, though the framework’s diagnostic power comes from holding all three in view together.

On governance, AI raises the question of accountability under conditions of algorithmic opacity: when an AI system makes a consequential error, who is responsible can no longer be assumed to have an obvious answer, and the European Union's AI Act has now made documented accountability, human oversight, and risk management legally binding for high-risk applications (48). Algorithmic accountability is a governance condition, not a compliance question, and the cost of failing it is paid in social licence (49–51), Section 7 operationalises the diagnostic this requires.

On environmental responsibility, AI raises the question of compute carbon: training and operating large models consumes energy at a scale now material to organisational emissions reporting, and sport organisations with net-zero commitments cannot exclude AI compute from that reporting without the commitment becoming cover rather than substance (52, 53).

On equity and access, AI raises the question of who is excluded by design: models trained on historical data reproduce the exclusions that have determined who participates and who advances, and tools that extract data from under-resourced participants without commensurate return export the value participation creates to entities that did not contribute to it, a pattern named data colonialism (54). AI in sport is equitable only when design choices — bias audits, representation requirements, price floors, data-return mechanisms — make it so; Section 7 sets out the diagnostic in full.

The section closes by returning to the three general-management models it began with, now sharpened by AI. The triple bottom line treats social and environmental outcomes as deliverables parallel to economic ones; in sport under AI they are neither parallel nor deliverables but enabling conditions whose strain turns existential the moment AI is deployed at scale. Creating shared value treats legitimacy as something strategic intent can manufacture; in sport under AI, legitimacy remains an outcome that strategic intent can forfeit but never simply produce, and shared value stays derivative — there is no shared value to share unless the contest is credible, the event is fair, and the AI is accountable. ESG supplies a reporting vocabulary, not the conditions that vocabulary is meant to describe; the metric can be satisfied while the condition fails, and AI is the technology in which that gap opens widest, because automated decision-making, compute emissions, and algorithmic bias erode the underlying conditions faster than any reporting cycle can register. The contribution of the architecture, stated in one line, is to hold legitimacy in its proper place: the outcome of the model working under defensible conditions, never an input the model can be instructed to deliver. Those conditions, in turn, do not arise in a vacuum — they are exerted upon, and reshaped by, a wider environment of forces, which the next section sets out.

**Proposition 4.** Legitimacy is an emergent outcome of the model operating under defensible governance, environmental, and equity conditions, not a deliverable that can be produced, purchased, or reported into existence; an AI deployment that satisfies a reporting metric while degrading the underlying condition accumulates legitimacy debt rather than legitimacy.

## 7 Context forces: the environment of value creation

The framework so far has described the endogenous structure of value creation in sport: three value types, four stakeholder groups, three enabling conditions, anchored in the irreducible activity. That structure does not sit in a vacuum. It operates inside an environment of exogenous forces that reshape who captures value, at what speed, on whose terms, and under which constraints. The framework treats these as context forces (Figure 3) rather than as components of the structure itself, because they change the operating conditions of value creation without changing the logic of it. Four force fields are consequential.

**Figure 3:**
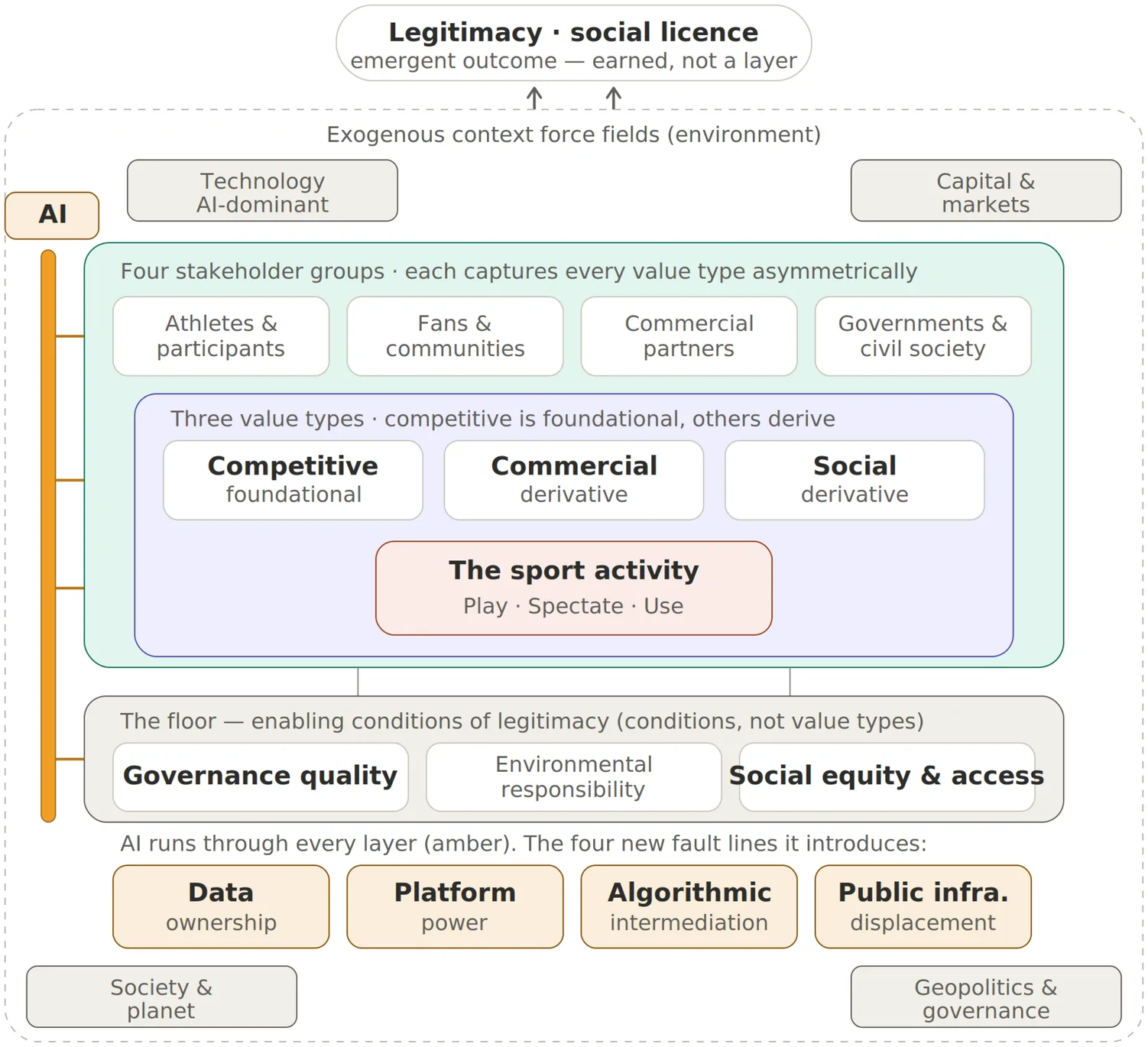
The sport value architecture (full system diagram). The Sport Value Architecture as a nested model: the irreducible sport activity at the core, three value types and four stakeholder groups around it, the three enabling conditions as the floor on which the structure rests, and the four context force fields as the surrounding environment. Artificial intelligence runs through every layer (shown in amber) and introduces the four structural tensions at the base. Legitimacy is the emergent outcome of the model working under defensible conditions — read from the model, not added as a fourth layer.

The first is technology, and in the current era technology is dominated by artificial intelligence. AI is the variable that has produced the most material change to the speed, scale, and distribution of value creation in sport over the past five years, and it will continue to be the dominant technological variable for at least the next decade. The framework treats the AI through-line as endogenous to the model where AI is deployed inside sport organisations, and exogenous where AI development sits outside the sport sector but reshapes what sport organisations can and must do. This distinction matters for governance: sport organisations have agency over the AI they deploy and limited agency over the AI being deployed against them by adjacent industries — gambling, betting, digital media platforms, sovereign actors. Both surfaces require attention; the framework supplies a different diagnostic for each.

The second force field is capital and markets. Private equity has restructured ownership in football and elsewhere; multi-club ownership has converted single clubs into portfolio components; sovereign wealth funds have entered sport as a vehicle for geopolitical positioning; impact investors have entered sport as a vehicle for ESG-aligned returns. Each of these changes the capture pattern across the stakeholder grid. The framework's diagnostic is consistent: capital that strengthens the conditions of competitive value creates durable commercial value, capital that erodes those conditions creates transient commercial value and accumulates legitimacy debt against future returns.

The third force field is society and planet. Climate change is a direct operational threat to sport events, infrastructure, athlete welfare, and the natural environments in which outdoor sport takes place (55). Demographic change — ageing populations in some economies, youth bulges in others, urbanisation across all of them — reshapes the demand for and the supply of sport participation. Pandemic-type shocks have demonstrated the fragility of live-event-dependent commercial models. Mental health standards have raised the expected duty of care across athlete and staff populations. None of these forces can be addressed by sport organisations acting alone, but each of them reshapes the conditions under which value creation in sport remains legitimate.

The fourth force field is geopolitics and governance. Sportswashing as a strategy of regime legitimation operates across the boundary between domestic and international politics (35, 37). Regulatory change in gambling, broadcasting ownership, competition law, and now AI is reshaping what can be lawfully done within sport business. International sanctions affect athlete eligibility, event hosting, and rights distribution. The formal role of sport in development policy — anchored in UN frameworks and operationalised through sport-for-development organisations — places sport inside foreign policy as well as inside social policy (56).

These four force fields shape the environment in which the framework operates. They do not change the framework. The three value types remain logically distinct. The four stakeholder groups remain the structural carriers of capture. The three enabling conditions remain the floor on which value creation rests. What the context forces change is the speed, the scale, and the terms on which the underlying structure is enacted. Distinguishing exogenous force from endogenous structure protects the framework from collapsing under each new cycle of technological, financial, or geopolitical change, and it preserves the framework's diagnostic value across changes that the model itself did not predict. The political and soft-power effects discussed in §3.5 become visible in the interaction between context forces — particularly geopolitics — and the credibility of the contest and the configuration of stakeholders. AI accelerates the interaction; it does not create it.

## 8 The AI through-line: a diagnostic apparatus

The argument advanced in the preceding sections can be summarised in a single move. Artificial intelligence is not located inside one part of the Sport Value Architecture. It runs through every part of it simultaneously. It generates value within each of the three value types: AI strengthens competitive value when it protects the integrity conditions of the contest and erodes competitive value when it compromises them; AI generates commercial value at finer granularity than any prior technology and directs capture toward those who own the data, control the algorithms, and retain the credibility of the contest; AI generates social value when its design choices are deliberate and verifiable and consumes social value when those design choices are absent. It reshapes capture across every stakeholder group. It stresses every enabling condition. And it presses on the irreducible activity itself, forcing the framework to specify what makes a contest count as a contest. The framework's conceptual contribution to the AI debate in sport is not a new layer; it is the integration. Holding every component of the system in view at once is the analytical move on which any defensible AI decision in sport now depends.

The framework supplies the apparatus to operationalise that integration. Six questions, asked in order, locate any AI deployment inside the system and identify where it is creating value, where it is consuming value, and where it is accumulating legitimacy debt.

The first question is on competitive value. *Does the AI deployment protect or improve the integrity, authenticity, and uncertainty of the contest itself, and the stakes attached to its outcome?* Performance analytics, injury prevention, scouting that expands the pool of identified talent, and officiating applied to clear factual questions can strengthen the integrity conditions. Synthetic athletes, fabricated footage, opaque selection systems, and gambling-integrated content erode them. AI that fails this question does not create competitive value. It consumes it.

The second question is on commercial value. *Who captures the commercial value the AI creates, and does the organisation deploying the AI own the data that makes the value possible?* AI-driven monetisation directs capture toward those who own the underlying data assets and the algorithms that act on them. Sport organisations that deploy AI without a defensible data position transfer the resulting commercial value to the platforms that supply the AI. The diagnostic is not whether the AI generates revenue. It is whether the revenue accrues to actors whose participation is necessary to the sport, or to intermediaries whose participation is not.

The third question is on social value. *Does the AI deployment expand or restrict access — to participation, to talent pathways, to coaching, to fandom, to the sport itself?* AI that democratises analytical capability previously confined to the well-resourced creates social value. AI that prices, sorts, or selects in ways that systematically exclude the populations on whose behalf the social-value claim is made consumes it. The diagnostic question is concrete and answerable: whose access is expanded by the AI, and whose access is restricted?

The fourth question is on governance. *Who is accountable when the AI is wrong?* The question must be answered before deployment, not constructed afterwards. High-risk AI applications — selection, medical diagnosis, disciplinary determination, fan data use, dynamic pricing — require documented accountability structures, human-in-the-loop review, and demonstrable risk management against the regulatory architecture the European Union's AI Act now establishes for high-risk systems (48). Algorithmic accountability is not a compliance question. It is a governance condition (49).

The fifth question is on environmental responsibility. *What is the compute footprint of the AI deployment, and is it accounted for in the organisation's environmental commitments?* AI training and inference at scale are now material to organisational emissions. Sport organisations with net-zero commitments either include AI compute in Scope 3 reporting and require vendor disclosure of compute energy use, or accept that the commitment is reporting cover rather than substance (52, 53).

The sixth question is on equity and access at the level of design. *Whose data trained the model, who is systematically under-represented in its outputs, and what design choices make the deployment compatible with equity?* Bias audits, representation requirements in training data, structural price floors that protect community access, and data return mechanisms that prevent extraction without reciprocity are the design moves that operationalise the equity condition. AI in sport is equitable only when these are present (54).

The six questions are the operationalisation of the framework's logical structure. A deployment that fails any of the six is a deployment that is accumulating legitimacy debt — for the organisation that deploys it and, through the cumulative effect of many such deployments, for the sport itself. The framework provides significant diagnostic value by making an organisation's legitimacy debt observable. The early detection occurs well before a system failure tests and challenges the underlying social licence.

**Proposition 5.** An AI deployment in sport is net legitimacy-positive only if it advances or enhances value across all three value types and satisfies all six diagnostic questions; failure on any single question accumulates legitimacy debt regardless of the deployment's performance on the others.

## 9 Stress-testing the framework: grassroots, Global South, Indigenous sport under AI

The framework's portability is its second test, and the AI extension does not exempt it. The test has an explicit criterion: whether the framework's four components — the value-type ordering, the stakeholder grid, the enabling-conditions floor, and the context forces — continue to hold their logical relationships to one another outside the Western, professional-league reference frame the earlier sections default to. A model built from that reference frame alone would fail at the edges where its assumptions do not hold. The framework is anchored in the activity rather than in any particular commercial form, and the anchor is what allows the model to hold across contexts.

Grassroots and recreational sport produce real competitive value at low intensity, thin or absent commercial value, and dominant social value. The framework explains why grassroots sport is rationally publicly funded even without commercial return: the social-value mechanism operates whether or not the commercial mechanism is engaged. AI in this context can democratise — accessible analytics, low-cost coaching tools, mental wellness support, community analytics for funding applications — or it can concentrate, directing grassroots into algorithmic talent pipelines whose data is captured upstream and whose value capture happens elsewhere (28, 29). The framework's logical order holds; the design choices in AI deployment determine which outcome gains.

Global South contexts sharpen the diagnostic. By Global South I mean not a strictly geographic region but a structural position in the world economy — the countries and communities, concentrated across Africa, Latin America, South and Southeast Asia, and the Pacific, that sit peripherally in the global distribution of capital, infrastructure, and, increasingly, the computational capacity on which AI depends. Sport infrastructure is often state-led or NGO-led; commercial markets are thinner; social-value claims carry political weight; mega-events are sometimes deployed for legitimation (32, 56). AI infrastructure is concentrated in the Global North, and the framework predicts what the resulting asymmetry costs. Data extracted from Global South sport ecosystems may not generate value that returns to those ecosystems; the value-capture pattern under AI is the contemporary form of data colonialism (54). The legitimacy-as-outcome point becomes more sharply diagnostic here: the social licence of imported AI tools is conditional on data return and design participation, and AI deployment without those conditions is legitimacy debt accumulated against host populations who had no part in the design.

Indigenous and traditional sport configurations push the framework toward its limit, and the check here draws on scholarship from within those communities rather than applying the model to them from outside. Indigenous sport scholars have documented sport and physical activity as an exercise of sovereignty in its own right — cultural, political, and spiritual citizenship enacted through community, relationality, strength, abundance, and resilience — rather than as derivative of the commercial or even the competitive form the framework treats as its anchor elsewhere (57). Community-based research with Indigenous youth similarly finds that participation in traditional games builds cultural pride, connection to Elders and land, and personal development through mechanisms that do not pass through a commercialised contest at all (58). Read against that scholarship, the framework holds only if its anchor is stated correctly: it is the sport activity, not competitive value specifically, that Indigenous and traditional configurations confirm as irreducible — the same distinction drawn in Section 3.4. Where the contest is ritual, identity-bound, and non-marketised, AI may be welcomed for specific purposes — heritage preservation, language preservation, accessible coaching — and rejected for others — commercialisation, surveillance, data extraction. The framework holds and clarifies which AI deployments are compatible with which value configurations: AI that supports the integrity of the activity and the conditions of equitable participation can strengthen Indigenous and traditional sport; AI that imports a commercial logic the configuration has refused dissolves the very value the configuration produces. Across all three stress tests the logical order holds, the stakeholder grid maps with different weightings, the enabling conditions remain conditions, and AI sharpens the diagnostic without changing the structure of the model.

## 10 Implications

The implications of the framework follow directly from its conceptual structure.

For theory, the framework supplies a common vocabulary across the disciplinary traditions that have until now studied sport value in fragmented and often non-communicating literatures. Sport economics, sport-for-development, sport governance, sport sociology, and sport management approach sport value from different traditions and arrive at definitions that do not commensurate. The framework does not collapse those differences; it makes them legible inside a single structure, so that the field's fragmentation can be diagnosed as a fragmentation of which value is being studied, rather than mistaken for fundamental disagreement.

For practice, the framework supplies a strategy and governance tool. The six-question diagnostic operationalises the framework for any AI decision in sport. The 4 × 3 matrix and its AI-extended version operationalise it for any decision that allocates value across stakeholders. The enabling conditions operationalise it for any decision that touches governance, sustainability, or equity. The framework does not adjudicate trade-offs, and it does not resolve them in advance: whether a given governance reform is substantive rather than cosmetic, or whose account of legitimacy should prevail in a contested case, remains a matter of judgement on which reasonable practitioners will disagree. What the framework forces is that the judgement be made explicitly, by named actors, with defensible justifications, rather than left implicit or asserted by default. That is the contribution practitioners need.

For policy, the framework supplies a basis for distinguishing legitimate from illegitimate claims on sport's public funding and social licence. Public investment in sport is justified by social value; social-value claims must be defensible, distributable, and verifiable; AI-mediated social-value claims must additionally satisfy the equity, governance, and sustainability conditions on which the social licence under AI conditions now depends. Policy that holds sport organisations to this standard will produce sustainable value; policy that does not will subsidise legitimacy debt.

For ethics, the framework makes the distributional question unavoidable. Sport ethics is not separate from sport strategy or sport AI policy; it is the same question — who captures what, at whose expense, under what conditions — asked in normative terms. The framework supplies the structure in which the ethical question can be posed concretely and answered accountably, in the language strategy and governance already use.

For future research, the framework's components are stated at a level of abstraction that invites empirical operationalisation rather than precluding it. The three value types can be operationalised as measurable constructs: competitive value through indicators of contest uncertainty and rule-enforcement consistency, commercial value through capture-share analysis across the stakeholder grid, and social value through the participation and wellbeing indicators already established in the sport-for-development literature. The enabling-conditions floor lends itself to comparative case designs that hold value creation constant while varying governance, environmental, and equity conditions, and to before-after designs around specific AI deployments that test whether the six-question diagnostic predicts subsequent legitimacy outcomes such as sponsor withdrawal, participation decline, or regulatory intervention. The propositions above are stated in a form suitable for that kind of testing, not merely as conceptual assertions.

## 11 Conclusion

Sport generates three logically distinct types of value: competitive, commercial, and social. They are captured asymmetrically across four stakeholder groups, anchored in an irreducible sport activity that is accessible through three modes, conditioned by three enabling conditions, and located within four exogenous force fields. Legitimacy is the outcome of the model working under defensible conditions, not a fourth type of value. The framework integrates these components into a single conceptual structure — the Sport Value Architecture — and supplies the diagnostic apparatus to apply it.

Artificial intelligence is the test under which the framework now has to work. AI runs through every component of the system simultaneously. It does not require a new model; it requires the existing model to be held entirely in view at the moment of each decision. The framework's six-question diagnostic operationalises that requirement. The four-by-three matrix, extended for AI, exposes the redistribution of capture that the technology produces. The treatment of legitimacy as outcome holds the line that AI cannot deliver and that AI cannot be allowed to consume.

The framework does not measure value, adjudicate trade-offs, or predict outcomes. It clarifies the conceptual terrain on which all of those tasks — including AI governance, strategy, and ethics in sport — must be conducted. Future work can measure, model, and case-study within this structure. The contribution is the clarity itself.

## References

[1] Westerbeek HM. Algorithmic fandom: how generative AI is reshaping sports marketing, fan engagement, and the integrity of sport. Front Sports Act Living. (2025) 7:1597444. 10.3389/fspor.2025.1597444 40406423 PMC12094936

[2] Westerbeek HM Van Schaik T. Platform power, athlete branding, generative AI, and the future of sport governance—a systematic review. Front Sports Act Living. (2025) 7:1642180. 10.3389/fspor.2025.1642180 41031328 PMC12477720

[3] Elkington J. Cannibals with forks: the triple bottom line of 21st century business. Capstone. (1997) 8:37–51. 10.1002/tqem.3310080106

[4] Elkington J. 25 Years ago I Coined the Phrase “triple Bottom Line.” Here’s why It’s Time to Rethink it. Boston: Harvard Business Review (2018). Available online at: https://hbr.org/2018/06/25-years-ago-i-coined-the-phrase-triple-bottom-line-heres-why-im-giving-up-on-it (Accessed May 08, 2026).

[5] Porter ME Kramer MR. Creating shared value. Harv Bus Rev. (2011) 89(1/2):62–77. 10.1007/978-94-024-1144-7_16

[6] Crane A Palazzo G Spence LJ Matten D. Contesting the value of “creating shared value.” Calif Manage Rev. (2014) 56(2):130–53. 10.1525/cmr.2014.56.2.130

[7] Dembek K Singh P Bhakoo V. Literature review of shared value: a theoretical concept or a management buzzword? J Bus Ethics. (2016) 137(2):231–67. 10.1007/s10551-015-2554-z

[8] Westerbeek HM. Why a value creation focus is critical for sport business growth [Blog post]. hanswesterbeek.com (2022). Available online at: https://www.hanswesterbeek.com/news/why-a-value-creation-focus-is-critical-for-sport-business-growth (Accessed April 16, 2026).

[9] Westerbeek HM Karg A. International Sport Business: Current Issues, Future Directions. London: Routledge (2022). 10.4324/9780429316043

[10] Woratschek H Horbel C Popp B. The sport value framework — a new fundamental logic for analyses in sport management. Eur Sport Manag Q. (2014) 14(1):6–24. 10.1080/16184742.2013.865776

[11] Vargo SL Lusch RF. Evolving to a new dominant logic for marketing. J Mark. (2004) 68(1):1–17. 10.1509/jmkg.68.1.1.24036

[12] Vargo SL Lusch RF. Institutions and axioms: an extension and update of service-dominant logic. J Acad Mark Sci. (2016) 44(1):5–23. 10.1007/s11747-015-0456-3

[13] Dowling M Leopkey B Smith L. Governance in sport: a scoping review. J Sport Manag. (2018) 32(5):438–51. 10.1123/jsm.2018-0032

[14] Freeman RE. Strategic Management: A Stakeholder Approach. Boston: Pitman (1984).

[15] Freeman RE Martin K Parmar B. Stakeholder capitalism. J Bus Ethics. (2007) 74(4):303–14. 10.1007/s10551-007-9517-y

[16] Westerbeek HM. The Enhanced Games, big pharmaceutical and protecting sport [Blog post]. hanswesterbeek.com (2026). Available online at: https://www.hanswesterbeek.com/news/the-enhanced-games-big-pharmaceutical-and-protecting-sport (Accessed April 16, 2026).

[17] Brandenburger AM Stuart HW. Value-based business strategy. J Econ Manag Strategy. (1996) 5(1):5–24. 10.1111/j.1430-9134.1996.00005.x

[18] Bowman C Ambrosini V. Value creation versus value capture: towards a coherent definition of value in strategy. Br J Manag. (2000) 11(1):1–15. 10.1111/1467-8551.00147

[19] Rottenberg S. The baseball players’ labor market. J Polit Econ. (1956) 64(3):242–58. 10.1086/257790

[20] Neale WC. The peculiar economics of professional sports. Q J Econ. (1964) 78(1):1–14. 10.2307/1880543

[21] Szymanski S. The economic design of sporting contests. J Econ Lit. (2003) 41(4):1137–87. 10.1257/jel.41.4.1137

[22] Westerbeek HM. Fans deserve fair play in major sports, not cheating and corruption. The Conversation (2016). Available online at: https://theconversation.com/fans-deserve-fair-play-in-major-sports-not-cheating-and-corruption-58999 (Accessed April 16, 2026).

[23] Smith ACT Westerbeek HM. The Sport Business Future. London: Palgrave Macmillan (2004). 10.1057/9780230513693

[24] Thompson A Naraine ML Parent MM. Exploring the nexus of digital technology and organizational change in non-profit sport organizations. Sport Management Review. (2024) 27(5):661–81. 10.1080/14413523.2024.2346633

[25] Westerbeek HM. Athletes are no longer just players: How digital platforms and AI are shifting power away from sport organisations [Blog post]. hanswesterbeek.com (2025). Available online at: https://www.hanswesterbeek.com/news/athletes-are-no-longer-just-players-how-digital-platforms-and-ai-are-shifting-power-away-from-sport-organisations (Accessed April 16, 2026).

[26] Herold E Singh A Feodoroff B Breuer C. Data-driven message optimization in dynamic sports media: an artificial intelligence approach to predict consumer response. Sport Management Review. (2024) 27(5):793–816. 10.1080/14413523.2024.2372122

[27] Westerbeek HM. The capital–credibility paradox: Why sport’s next big test is about trust, not cash [Blog post]. hanswesterbeek.com (2025). Available online at: https://www.hanswesterbeek.com/news/the-capitalcredibility-paradox-why-sports-next-big-test-is-about-trust-not-cash (Accessed April 16, 2026).

[28] Westerbeek HM Eime R. The physical activity and sport participation framework — a policy model toward being physically active across the lifespan. Front Sports Act Living. (2021) 3:608593. 10.3389/fspor.2021.608593 34027402 PMC8138121

[29] Eime R Charity M Westerbeek HM. The sport participation pathway model (SPPM): a conceptual model for participation and retention in community sport. Int J Sport Policy Politics. (2022) 14(2):291–304. 10.1080/19406940.2022.2034913

[30] Coalter F. A Wider Social Role for Sport: Who’s Keeping the Score? London: Routledge (2007).

[31] Schulenkorf N Sherry E Rowe K. Sport for development: an integrated literature review. J Sport Manag. (2016) 30(1):22–39. 10.1123/jsm.2014-0263

[32] Darnell SC Chawansky M Marchesseault D Holmes M Hayhurst L. The state of play: critical sociological insights into recent “sport for development and peace” research. Int Rev Sociol Sport. (2018) 53(2):133–51. 10.1177/1012690216646762

[33] Smith ACT Westerbeek HM. Sport as a vehicle for deploying corporate social responsibility. J Corp Citizensh. (2007) 25:43–54. 10.9774/GLEAF.4700.2007.sp.00007

[34] Robertson J Eime R Westerbeek HM. Community sports clubs: are they only about playing sport, or do they have broader health promotion and social responsibilities? Ann Leis Res. (2018) 22(2):215–32. 10.1080/11745398.2018.1430598

[35] Westerbeek HM Spaaij R. Boycotts in sport may not advance human rights. But they do harm individual athletes. The Conversation (2022). Available online at: https://theconversation.com/boycotts-in-sport-may-not-advance-human-rights-but-they-do-harm-individual-athletes-185208 (Accessed April 16, 2026).

[36] Grix J Dinsmore A Brannagan PM. Unpacking the politics of “sportswashing”: it takes two to tango. Politics. (2023) 45(3):377–98. 10.1177/02633957231207387

[37] Grix J Brannagan PM. Sports mega-events as foreign policy: sport diplomacy, “soft power,” and “sportswashing.”. Am Behav Sci. (2024). 10.1177/0002764224126204240809922

[38] Harrison JS Wicks AC. Stakeholder theory, value, and firm performance. Bus Ethics Q. (2013) 23(1):97–124. 10.5840/beq20132314

[39] Boltanski L Thévenot L. On Justification: Economies of Worth (C. Porter, Trans.). Princeton: Princeton University Press (2006). (Original work published 1991).

[40] Stark D. The Sense of Dissonance: Accounts of Worth in Economic Life. Princeton: Princeton University Press (2009).

[41] Srnicek N. Platform Capitalism. Cambridge: Polity Press (2017).

[42] Suchman MC. Managing legitimacy: strategic and institutional approaches. Acad Manag Rev. (1995) 20(3):571–610. 10.5465/amr.1995.9508080331

[43] Deephouse DL Bundy J Tost LP Suchman MC. Organizational legitimacy: six key questions. In: Greenwood R Oliver C Lawrence TB Meyer R, editors. The SAGE Handbook of Organizational Institutionalism. 2nd edn. London: Sage (2017). p. 27–54.

[44] Moffat K Zhang A. The paths to social licence to operate: an integrative model explaining community acceptance of mining. Resour Policy. (2014) 39:61–70. 10.1016/j.resourpol.2013.11.003

[45] Gehman J Lefsrud LM Lounsbury M. Social license to operate: legitimacy by another name? Can Public Adm. (2017) 60(2):293–317. 10.1111/capa.12218

[46] Geeraert A van Eekeren F. Good Governance in Sport: Critical Reflections. London: Routledge (2022).

[47] Chappelet J-L Mrkonjic M. Basic Indicators for Better Governance in International Sport (BIBGIS): An Assessment Tool for International Sport Governing Bodies (IDHEAP Working Paper 1/2013). Lausanne: Swiss Graduate School of Public Administration (2013).

[48] European Parliament & Council of the European Union. Regulation (EU) 2024/1689 of the European Parliament and of the Council of 13 June 2024 laying down harmonised rules on artificial intelligence (Artificial Intelligence Act). Official Journal of the European Union, L, 2024/1689 (2024). Available online at: http://data.europa.eu/eli/reg/2024/1689/oj (Accessed April 16, 2026).

[49] Eubanks V. Automating Inequality: How High-tech tools Profile, Police, and Punish the Poor. St. New York: Martin’s Press (2018).

[50] Kim J-H Kim J Kang H Youn B-Y. Ethical implications of artificial intelligence in sport: a systematic scoping review. J Sport Health Sci. (2025) 14:101047. 10.1016/j.jshs.2025.101047 40316133 PMC12226371

[51] Floridi L Cowls J. A unified framework of five principles for AI in society. Harv Data Sci Rev. (2019) 1(1):2–14. 10.1162/99608f92.8cd550d1

[52] Strubell E Ganesh A McCallum A. Energy and policy considerations for deep learning in NLP. In: Korhonen A Traum D Màrquez L, editors. Proceedings of the 57th Annual Meeting of the Association for Computational Linguistics. Florence: Association for Computational Linguistics (2019). p. 3645–50. 10.18653/v1/P19-1355

[53] Patterson D Gonzalez J Le Q Liang C Munguia L-M Rothchild D. Carbon emissions and large neural network training. (2021). Available online at: https://arxiv.org/abs/2104.10350 (Accessed April 24, 2026).

[54] Couldry N Mejias UA. The Costs of Connection: How Data is Colonizing Human Life and Appropriating it for Capitalism. Stanford: Stanford University Press (2019).

[55] Orr M Inoue Y. Sport versus climate: introducing the climate vulnerability of sport organizations framework. Sport Manag Rev. (2018) 22(4):452–63. 10.1016/j.smr.2018.09.007

[56] Lindsey I Darby P. Sport and the sustainable development goals: where is the policy coherence? Int Rev Sociol Sport. (2019) 54(7):793–812. 10.1177/1012690217752651

[57] Ali-Joseph A Leonard K Welch NM. Indigenizing sport research: analyzing protective factors of exercising sovereignty in North America. Sociol Sport J. (2023) 40(4):410–8. 10.1123/ssj.2023-0006

[58] Dubnewick M Hopper T Spence JC McHugh T-LF. There’s a cultural pride through our games”: enhancing the sport experiences of Indigenous youth in Canada through participation in traditional games. J Sport Soc Issues. (2018) 42(4):207–26. 10.1177/0193723518758456

